# Evaluating Polo Helmet Performance Across Different Impact Test Systems

**DOI:** 10.1007/s10439-025-03731-0

**Published:** 2025-04-17

**Authors:** Nicole E.-P. Stark, Mark T. Begonia, Steve Rowson

**Affiliations:** 1 Department of Biomedical Engineering and Mechanics, Virginia Tech, 325 Stanger St., Kelly Hall 120, Blacksburg, VA 24061 USA; 2 Institute for Critical Technology and Applied Science, Virginia Tech, Blacksburg, VA USA

**Keywords:** Helmet testing, Polo helmets, Rotation, NOCSAE, Oblique, Pendulum

## Abstract

**Purpose:**

This study evaluated head impact response between different helmet impact test systems by comparing the performance of ten polo helmets.

**Methods:**

Helmets were evaluated using three test systems: a twin-wire guided drop tower, an oblique drop tower, and an impact pendulum. Impact tests were conducted at matched locations (front boss, side, rear boss) and speeds (3.46, 5.46 m/s). We employed a linear mixed model with helmet model as a random effect and calculated the least square mean differences between systems for peak linear acceleration (PLA), peak rotational acceleration (PRA), peak rotational velocity (PRV), and concussion risk. Correlations between systems by impact speed were explored, using linear models of each system as a function of the others, and calculated Spearman rank correlation coefficients between test systems for each dependent variable.

**Results:**

Our results found distinct differences in PRA and concussion risk between the oblique and the pendulum impact systems due to the driving force. The acceleration range across helmet models was substantial, and responses differed between test systems at matched impact conditions. However, there were similarities between test systems in the rank order of helmet models. Head acceleration differences between helmets translated to larger differences in concussion risk between helmet models.

**Conclusion:**

These trends provide a framework for comparing the headform’s response across varying loading conditions. When selecting a test system to evaluate helmets for a specific sport, it is essential to consider the relevant impact conditions and loading patterns to ensure that laboratory tests accurately represent real-world scenarios.

## Introduction

The U.S. Polo Association (USPA) was established in 1890 to govern the sport of polo in the United States. However, polo is an international sport that is played in over 80 countries [1]. In 2015, the Hurlingham Polo Association (HPA), Great Britain's governing body, had nearly 3000 players and 13,000 horses registered [1]. Polo is considered a high-risk sport, with injuries occurring due to falls, collisions, or impacts from mallets and balls. The injury rate in polo is approximately 7.8/1000 hr of play, which is lower than other contact sports such as soccer (16.9/1000 hr) and rugby (44.9/1000 hr) [2–5]. However, 64% of injuries sustained by polo players are considered severe, with injuries that include bone fractures, ligament tears, and concussion [2]. Polo governing bodies now require players to wear helmets that meet approved testing standards for players to participate [6, 7].

All polo players must wear an approved helmet, but the standards can vary by region and organization. In the United States, helmet standards for polo and equestrian sports were developed by organizations such as the National Operating Committee on Standards for Athletic Equipment (NOCSAE), the American Society for Testing & Materials (ASTM), and the Snell Memorial Foundation. Similar standards were developed internationally through the Products Approval Specification (PAS) in Great Britain, the Joint Standards Australia/Standards New Zealand Committee (AS/NZS), and the European Committee for Standardization (CEN). These standards are labeled on the helmets to guide consumers [7–9]; however, when asked to identify factors that influence helmet selection, 49% of polo players considered appearance as the primary factor, while only 29% considered safety as the primary factor [1]. These standards encompass a range of impact velocities, locations, and speeds; however, for each standard, the headform is fixed, allowing only vertical translational motion. However, both linear and rotational accelerations are key predictors of brain injuries [10–14], and the current polo helmet testing methods solely evaluate linear acceleration.

Although oblique and pendulum helmet test rigs are commonly used for contact sports, few studies have directly compared different helmet testing systems. Studies comparing guided (fixed headform to a guided rail, not twin wire) impacts to free-fall impacts have found that guided falls produced, on average, higher linear accelerations, while free falls resulted in lower linear accelerations [15–18]. The reduction in linear acceleration during free-fall impacts is due to some impact energy being redirected into rotational acceleration. Another factor to consider is the reaction of components such as the ball arms that are attached to the monorail or twin-wire carriages. Further, guided impacts prevent headform rotation and are unable to evaluate rotationally induced brain injuries. Consequently, Meng et al. found that free-fall impacts resulted in up to a 93% increase in concussion risk compared to guided falls, measured by maximal principal strain [15]. However, free-fall impacts have been criticized for introducing helmet misalignment, especially with the increase in complex helmet designs, which can alter impact location, eccentricity, and repeatability [15, 16]. Another system commonly used to evaluate helmets is a pendulum impactor [19–21]. These test systems typically consist of a frame and a swinging arm with an attached impactor that strikes a helmet mounted on a headform, which is attached to a neck and representative torso mass. Pendulum impactor systems allow for repeatable impact speeds and can be tuned to sport-specific impact conditions using the principles of momentum transfer. Nonetheless, pendulum impacts have different constraints compared to guided and free-fall systems that could affect head kinematics response but have not yet been directly compared.

Given the severity of polo-related head injuries and the variety of polo helmet certification criteria, studies have evaluated the overall polo helmet performance. Transportation Research Laboratory (TRL) evaluated nine polo helmet performances under the Equestrian New Helmet Assessment Program (ENHAP) comprised pull-off, impact, and dynamic crush tests on multiple headform sizes [22]. A rating system was also implemented, with helmets receiving no more than two stars if peak headform accelerations exceeded 330 g or one star if peak headform accelerations exceeded 300 g and energy absorption was less than 69 J. During impact and crush testing, four helmets that employed a modern design (i.e., energy absorption layer) outperformed the five helmets without absorption layers. Furthermore, helmets with 3-point harnesses outperformed helmets with a traditional single-strap design [22]. USPA contracted the Southern Impact Research Center (SIRC) to assess the performance of several polo helmet models. Evaluations were conducted at ambient temperatures in accordance with both NOCSAE and ASTM standards. Based on findings by the SIRC, only six of the eleven helmets tested had complied with ASTM F1163, while none complied with NOCSAE 050 [23, 24]. The Research Institutes of Sweden (RISE) performed polo helmet evaluations, in 2021, on fifteen adult and youth-sized equestrian helmets and included guided drop and oblique impact tests [25]. Peak linear acceleration response differed between helmet models for both guided and oblique impacts, and peak rotational kinematics differed between helmet models for oblique impacts [25]. These studies further highlight the variability in the impact attenuation of polo helmets despite conforming to standards.

This study assesses how different test systems influence headform kinematic response by comparing the performance of 10 polo helmet models under various linear and rotational loading conditions. The test systems included the NOCSAE drop tower (1), an impact pendulum (2), and an oblique drop tower (3). We hypothesize that the pendulum would produce the lowest linear accelerations compared to the NOCSAE and the oblique system. Furthermore, we hypothesized that rotational kinematics would differ between systems for matched impact locations because of the way rotation is produced between test systems. These findings will highlight differences and draw comparisons between helmet impact testing systems.

## Methods

**Fig. 1:**
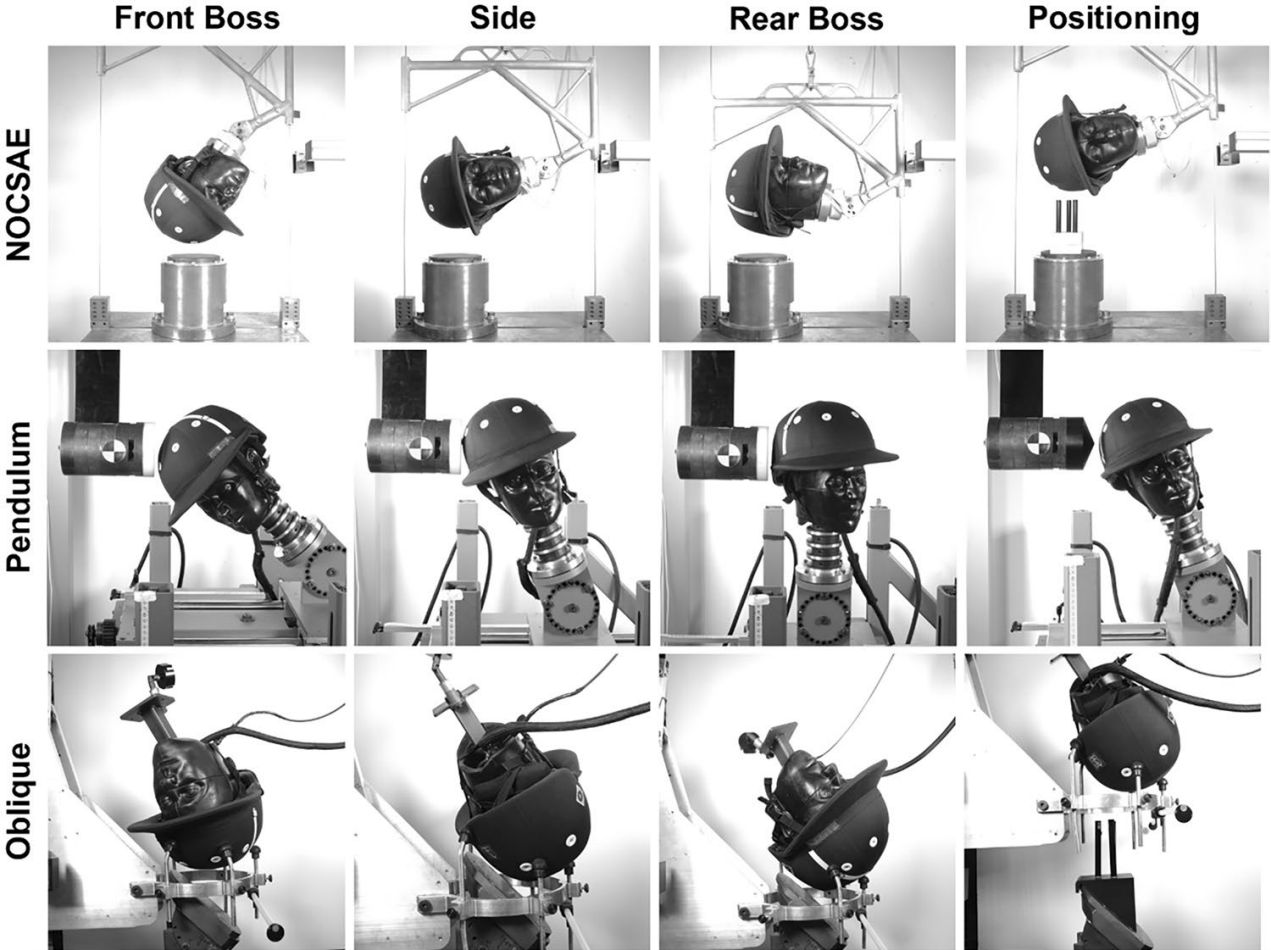
Matched impact locations were tested across the 3 test systems.

Our study aimed to compare the impact performance of 10 polo helmet models (Table 1) under varying linear and rotational loading conditions. Four helmet models comply with the current NOCSAE standard, and one helmet includes Multi-directional Impact Protection System (MIPS) technology. This study consisted of testing the helmets across three different test systems (pendulum impactor, oblique drop tower, and NOCSAE drop tower). We conducted a total of 180 impact tests, using three test systems, two impact speeds, and three impact locations (Fig. 1). We tested two impact speeds: 3.46 and 5.46 m/s, which align with the NOCSAE standard and represent low and high-impact severities. The impact locations included the front boss, side, and rear boss, chosen to enable matched impact locations for all three systems (Fig. 1; Table 2). Azimuth and elevation angles were collected following procedures described by Harlos et al., which describes a reliable and repeatable way to quantify impact location azimuth and elevation angles [26]. Each individual helmet was impacted only once per the three impact locations (front boss, side, and rear boss) on the left and right side of the helmet, for a total of six impacts on each individual helmet. The minimum separation distance between impact locations was 120 mm, following testing guidelines of the Consumer Product Safety Commission (CPSC).

**Table 1:**
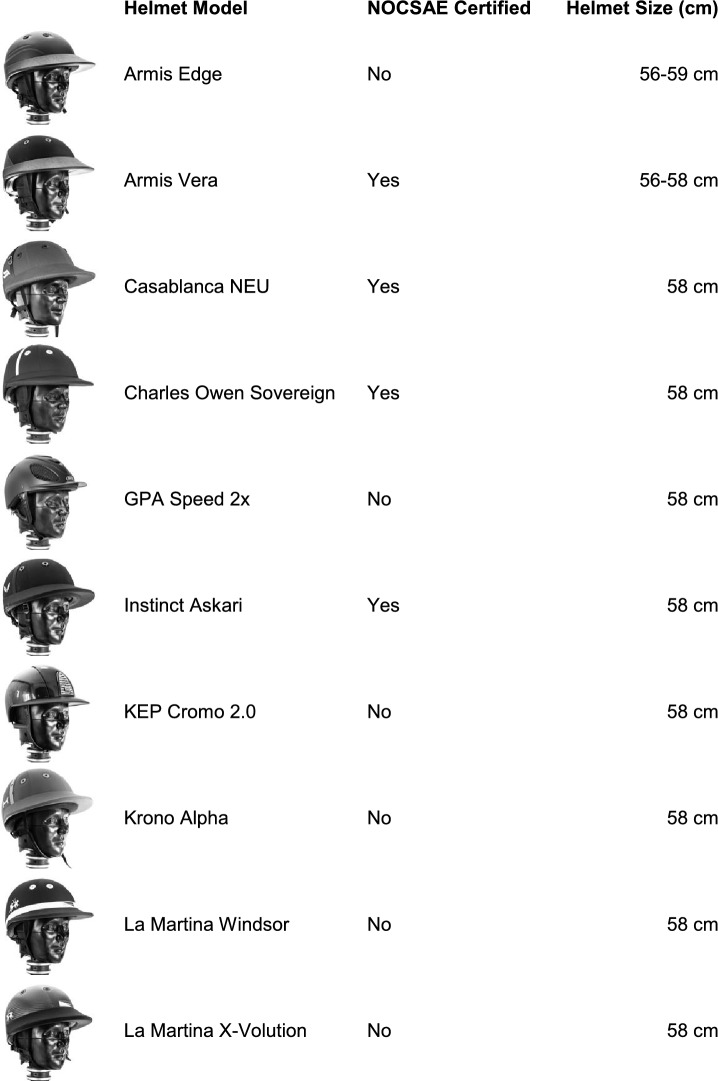
The 10 polo helmet models included in this test series

**Table 2:** Impact location azimuth and elevation angle used across the three impact systems

Location	Azimuth (°)	Elevation (°)
Front boss	53	68
Side	144	20
Rear boss	106	50

Azimuth angles are positive for right-side impacts and negative for left-side impacts.

### NOCSAE Drop Tower

The NOCSAE drop tower complies with the system specified in the current NOCSAE test standard (ND 001). It consists of a twin-wire guided drop with a carriage, positioning components (stem, rotator, and collar), and an instrumented NOCSAE headform. This assembly was dropped onto a flat anvil covered with a Modular Elastomer Programmer (MEP) pad.

Impact locations were defined first on the NOCSAE drop tower to ensure that standards-based helmet positions could be translated to the other two test systems, impact locations were positioned to ensure no interactions with the helmet visors across all helmet models. After adjusting the stem and rotator to achieve a predetermined impact location, we used a NOCSAE nose gauge to ensure a consistent distance between the headform nose and the brim of the helmet. The measurement was made using the nose tip, which is 20 mm below the basic plane. The distance between the headform nose and helmet brim was 80 mm, which was the same across all helmet models. Between each impact, we fully loosened the retention system, positioned the helmet on the headform, and then tightened the retention system. We then marked the three impact locations by using a custom mount that was attached to the flat anvil. This mount consisted of three vertical lasers that projected the impact location onto the helmet shell so that it could be marked and then used to define the corresponding impact location on the other two test systems (Fig. 1).

We measured linear head kinematics using a three-degree-of-freedom sensor package mounted at the NOCSAE headform CG. The sensor package consisted of three accelerometers (Endevco 7264B, 2000 g ± 1%, PCB Piezotronics, Depew, NY). Rotational acceleration was not captured for this system as the system constrains the headform’s rotation.

### Impact Pendulum

The pendulum impactor struck a helmeted NOCSAE headform mounted on a Hybrid III neck and sliding torso mass (16 kg), measuring both linear and rotational acceleration for direct head impacts. A flat nylon impactor face with an 8 in. diameter was attached to the pendulum arm [27]. A nylon impactor face was used as a best match impact surface to the NOCSAE MEP pad. The headform and neck assembly were mounted on a sliding mass representing the effective torso mass of a 50th-percentile male. This sliding mass was part of a target table (Biokinetics, Ottawa, Ontario, Canada) that allows linear and rotational head motion to be produced upon impact.

We set the impact locations on the pendulum to match the locations identified previously on the NOCSAE drop tower. The marked helmet was placed on the impact pendulum headform and then a NOCSAE nose gauge was used to maintain a consistent distance between the headform nose and helmet brim. A conical impactor face was attached to the pendulum arm, and the target table was adjusted so that the apex of the impactor face was aligned with the center of the helmet markings. We then replaced the conical impactor face with the flat nylon impactor face, applied chalk to its surface, and lightly contacted the helmet shell to visually confirm the first point of contact from the subsequent imprints.

We measured linear and rotational head kinematics using a six-degree-of-freedom sensor package mounted at the NOCSAE headform CG. The sensor package consisted of three accelerometers and a triaxis angular rate sensor (DTS ARS3 PRO, 18 k deg/s ± 2%, Diversified Technical Systems, Seal Beach, CA).

### Oblique Drop Tower

The oblique drop tower, on the other hand, had a helmeted NOCSAE head free-falling onto an angled anvil, measuring both linear and rotational acceleration for oblique angle impacts [28]. Impact tests were performed using a NOCSAE headform onto an adjustable steel anvil set at 40° (measured from the horizontal). This anvil configuration allowed the normal component of velocity to be matched to NOCSAE standard test velocities within the maximum allowable height of the oblique drop tower. The two normal component impact speeds were 3.46 and 5.46 m/s. A steel anvil was used to match better the surface of the MEP pad and nylon impactor face used on the other impact systems as compared to the 80-grit sandpaper typically used in oblique drop tower helmet testing.

For the oblique drop tower, we set the impact locations to match the locations from the marked helmet used on the NOCSAE drop tower. The marked helmet was positioned on the headform, and a NOCSAE nose gauge was used to ensure a consistent distance between the headform nose and helmet brim. A custom three-laser mount was positioned on the angle anvil, where the lasers projected the impact location onto the helmet surface. The helmeted NOCSAE headform was then positioned in a support ring. Impact locations were set and confirmed using a dual-axis inclinometer, cross-level laser, and wall-mounted grid to ensure consistent positioning on the support ring of the drop tower. The NOCSAE headform was instrumented with a six-degree-of-freedom sensor package that consisted of three accelerometers (Endevco 7264B-2000, PCB Piezotronics, Depew, NY), and a triaxis angular rate sensor (ARS) (ARS3 PRO, Diversified Technical Systems, Seal Beach, CA), mounted at the headform CG.

### Analysis

Acceleration and angular rate data were transformed using a rotation matrix to account for the offset angle of the sensor package when mounted at the NOCSAE headform CG. We sampled data at 20 kHz for all tests. We filtered kinematic signals using a four-pole phaseless digital Butterworth low pass filter in compliance with SAE J211 specifications. We applied a cutoff frequency of 1650 Hz to linear acceleration signals and 300 Hz to angular rate signals.

We computed rotational acceleration by differentiating angular rate signals using the five-point central difference method. We then calculated the resultant peak linear acceleration (PLA), peak rotational acceleration (PRA), and peak rotational velocity (PRV) for each test. To determine the overall severity of each impact and present the kinematics in the context of injury risk, we computed concussion risk from peak linear and rotational accelerations [10]. The injury risk function (Equation 1) was derived from a multivariate logistic regression analysis of data that was collected from football players instrumented with helmet-mounted sensors and then paired with diagnosed concussions [10]. The bivariate risk function considers both PLA ($a$) and PRA ($\alpha$) because linear and rotational components are often considered in assessments of injury risk [29–32].

1 $${\text{risk}}\left( {a,\alpha } \right) = \frac{1}{{1 + e^{{ - \left( { - 10.2 + 0.0433a + 0.000873\alpha - 0.00000092a\alpha } \right)}} }}$$

We carried out three types of analyses—a comparison of the time series and peak kinematics produced by each test system and an examination of helmet performance across these systems. Linear accelerations were compared across all three systems, and rotational accelerations and velocities between the impact pendulum and oblique drop tower. We compared the time series data by plotting the mean and 95% confidence intervals (CI) for resultant and axis-specific components of linear acceleration, rotational acceleration, and rotational velocity. We employed a linear mixed model with a helmet model as a random effect and calculated the least square mean differences between systems. We also explored relationships between systems by impact speed, using linear models of each system as a function of the others.

When contrasting peak kinematics and concussion risk between helmet models, we computed mean values and 95% CI by impact speed across systems and locations. We calculated Spearman rank correlation coefficients (rho) between test systems for each dependent variable averaged across test systems, impact locations, and impact speeds. All analyses were completed in R (Version 3.3.0, RStudio; Boston, Massachusetts, USA).

## Results

### Test System Comparison

#### Linear Acceleration

**Fig. 2:**
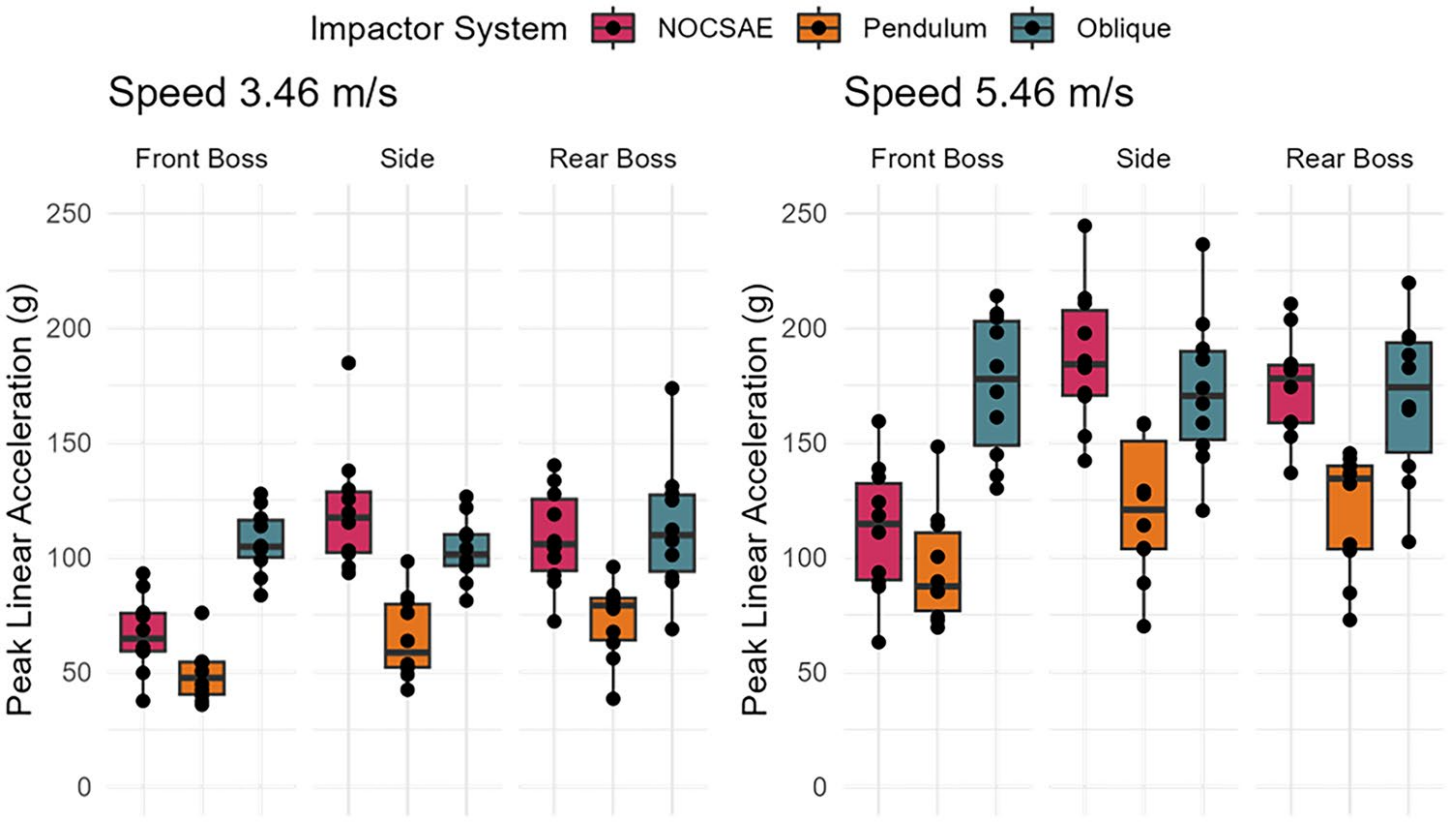
Distributions of peak linear accelerations between test system, impact location, and impact speed across all 10 helmets.

Test system (*p* < 0.0001), impact location (*p* < 0.0001), and impact speed (*p* < 0.0001) all influenced peak linear acceleration measures (Fig. 2). On average, the oblique drop tower produced linear accelerations 52.7 g [95% CI 44.5, 60.9 g] higher than the pendulum (*p* < 0.0001). The NOCSAE drop tower produced linear accelerations 40.9 g [32.7, 49.1 g] higher than the pendulum (*p* < 0.0001). The oblique drop tower produced linear accelerations 11.8 g [3.6, 20.0 g] higher than the NOCSAE drop tower (*p* = 0.0140).

At 3.46 m/s, there is a high correlation between the Pendulum and Oblique system and the Pendulum and NOCSAE system (Table 3). The comparison between Oblique and NOCSAE systems showed a lower adjusted R-squared of 0.104 (*p* = 0.0462), suggesting a weaker correlation. At the higher impact speed of 5.46 m/s, the relationship between Oblique and NOCSAE comparison at this speed showed a negative adjusted R-squared (− 0.017, *p* = 0.4781), indicating a lack of explanatory power. However, the Pendulum and Oblique systems had a strong correlation, and the Pendulum and NOCSAE systems had a moderate correlation.

**Table 3:** Adjusted R-squared values describing how much of the variance in one system is explained by the other system for Peak Linear Acceleration

Speed (m/s)	Model	Adj. R-squared	*p*-value
3.46	Oblique ~ Pendulum	0.327	0.0006
	Oblique ~ NOCSAE	0.104	0.0462
	NOCSAE ~ Pendulum	0.515	< 0.0001
5.46	Oblique ~ Pendulum	0.505	< 0.0001
	Oblique ~ NOCSAE	-0.017	0.4781
	NOCSAE ~ Pendulum	0.122	0.0340

**Fig. 3:**
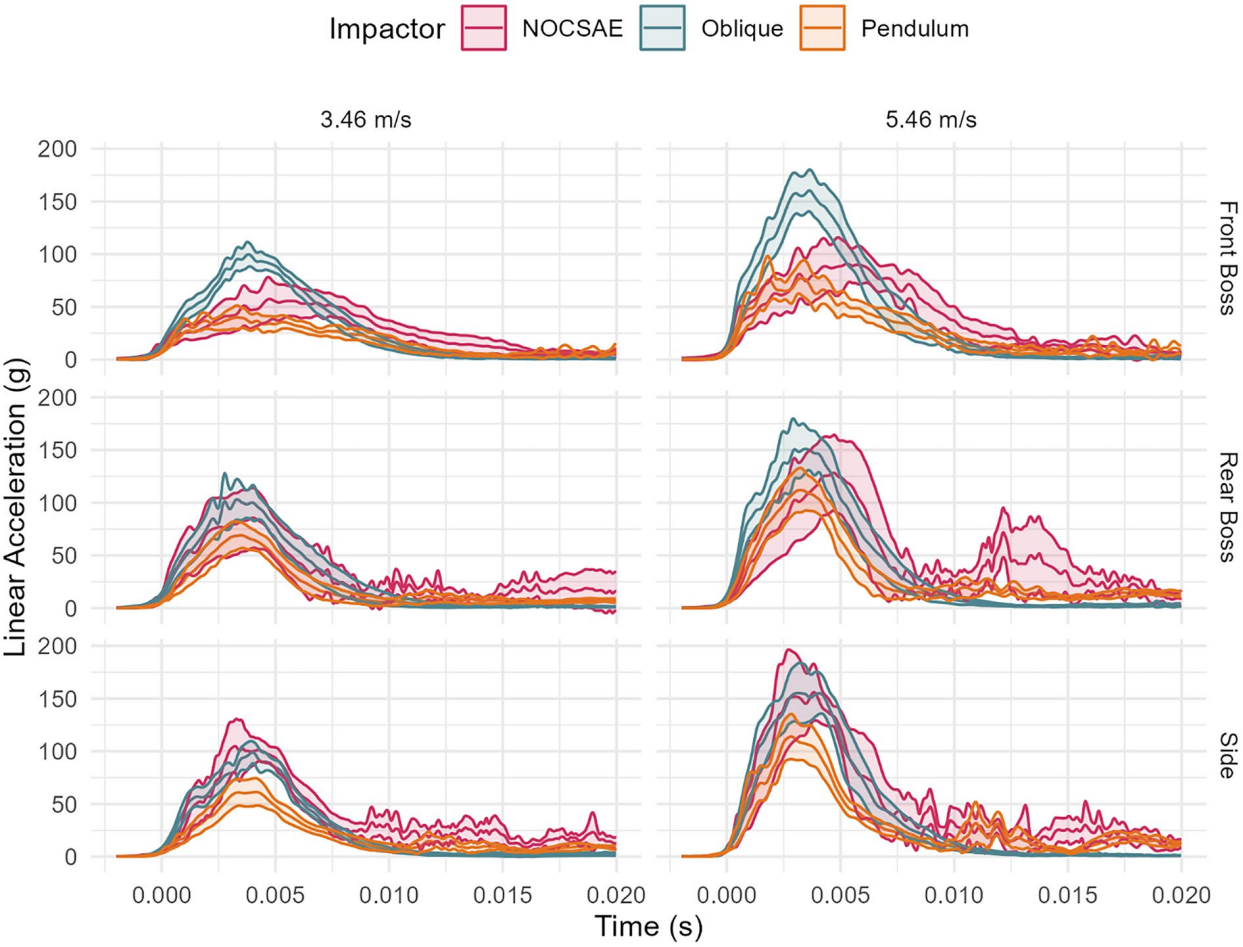
Linear acceleration time traces between systems and by location (mean and 95% confidence intervals). Pendulum impacts had lower linear acceleration values, but traces were similar between systems.

The linear impact response varied between test systems over the impact duration (Fig. 3). The pendulum system had lower resultant linear acceleration over a shorter duration compared to the other systems. Axis-specific trace comparison can be found in the Appendix (Figs. 9, 10, 11).

### Rotational Acceleration

**Fig. 4:**
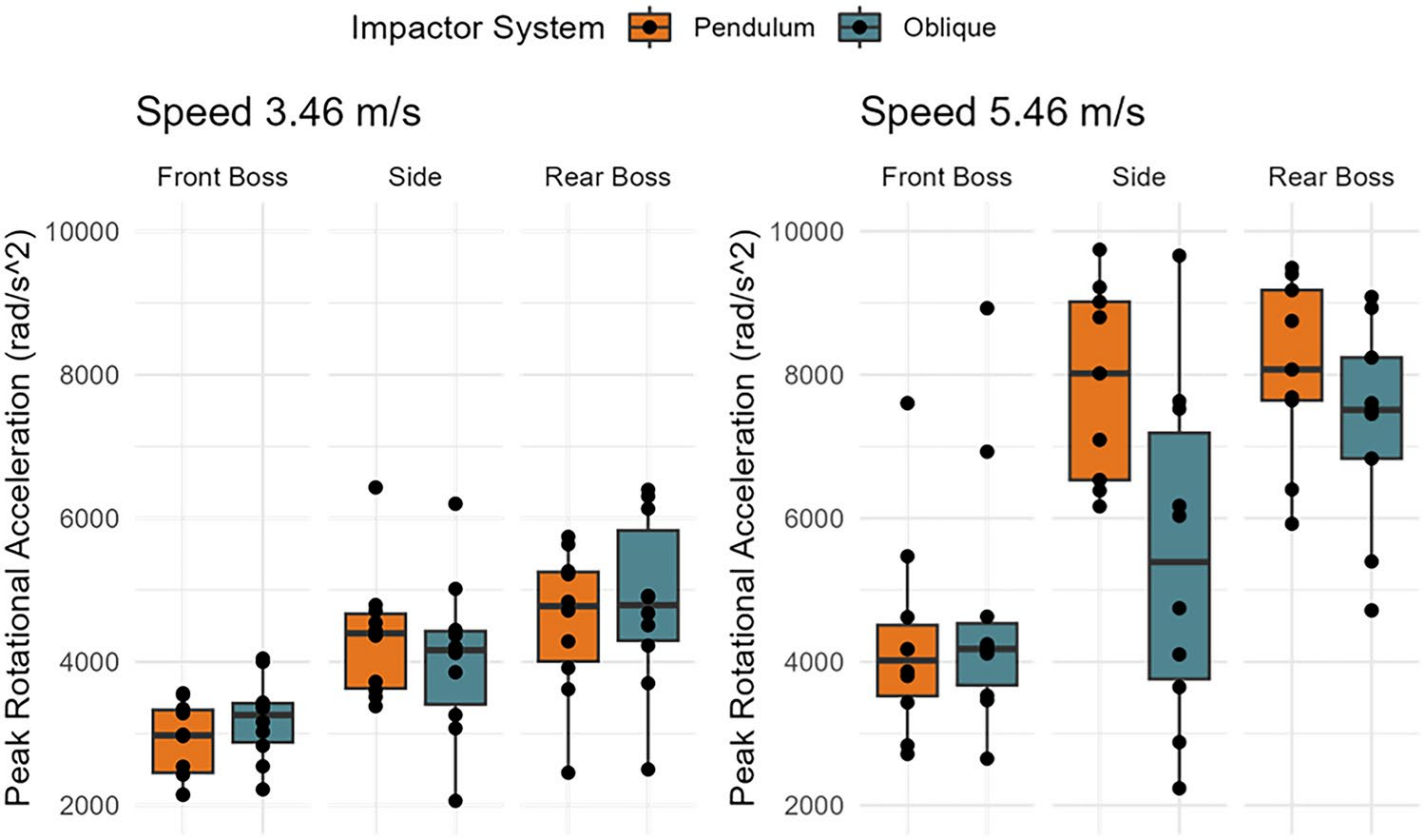
Distributions of peak rotational accelerations between test system, impact location, and impact speed across all 10 helmets.

Test system (*p* = 0.0546), impact location (*p* < 0.0001), and impact speed (*p* < 0.0001) influenced rotational acceleration measures to varying degrees (Fig. 4). On average, the oblique drop tower produced rotational accelerations 460 rad/s^2^ [ − 2, 923 rad/s^2^] higher than the pendulum impactor. Adjusted R-squared values from the linear models of the pendulum compared to the oblique system suggested moderate associations between test systems for rotational acceleration (Table 4).

**Table 4:** Adjusted R-squared values describing how much of the variance in one system is explained by the other system for Peak Rotational Acceleration

Speed (m/s)	Model	Adj. R-squared	*p*-value
3.46	Oblique ~ Pendulum	0.403	< 0.0001
5.46	Oblique ~ Pendulum	0.208	0.0066

**Fig. 5:**
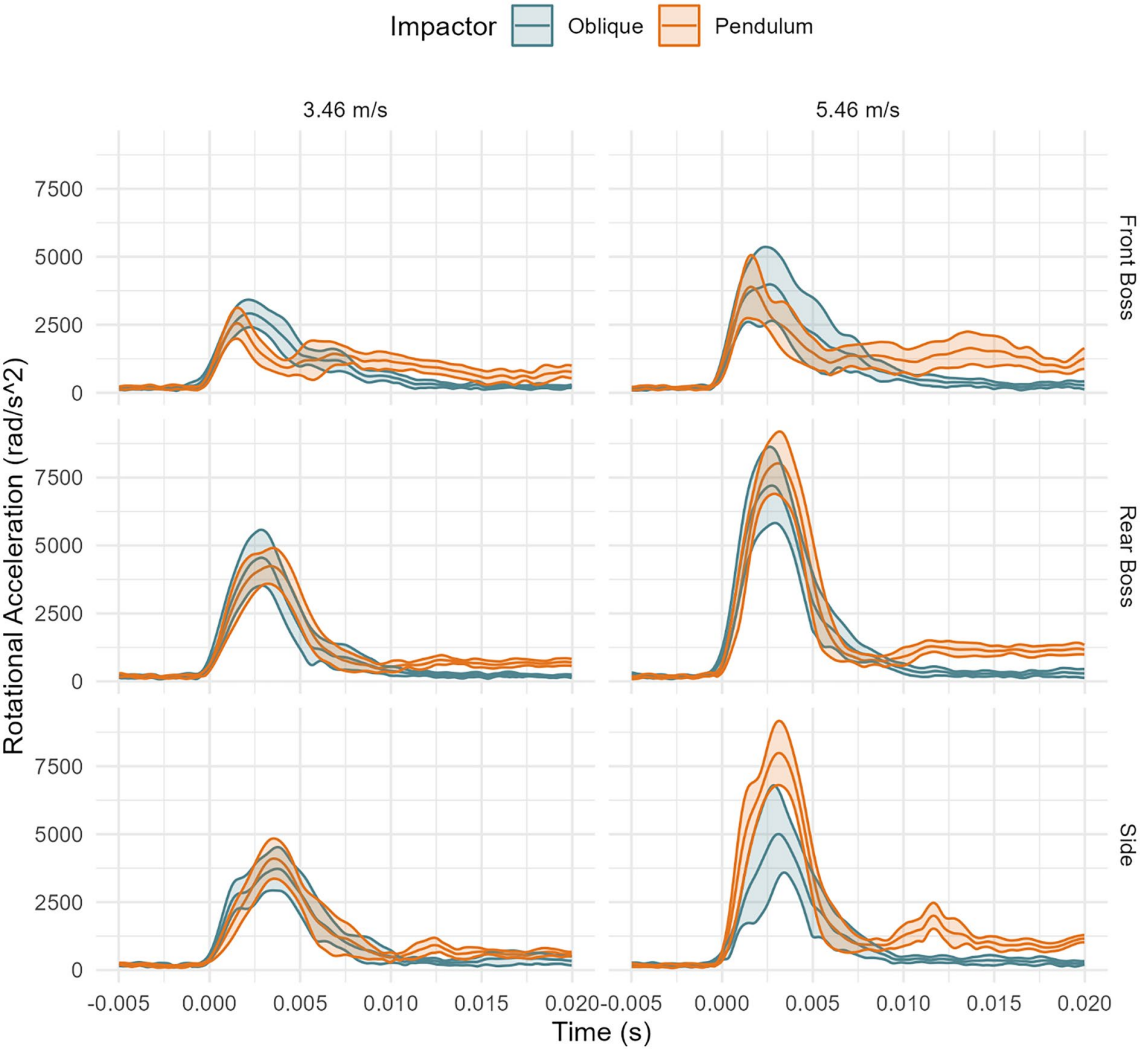
Rotational acceleration time traces between systems and by location (mean and 95% confidence intervals). Oblique impacts had higher rotational acceleration values at high-speed rear boss and side impacts, but traces were similar between systems.

The rotational impact response was similar between test systems over the impact duration (Fig. 5). The largest deviations between systems were seen for high-speed side impacts. However, when comparing axis-specific traces, the rear boss impacts had the largest variance across axis component traces (Appendix Figs. 12, 13, 14). The oblique drops had primary X and Z axis rotation while the pendulum had primarily X and Y rotations.

**Fig. 6:**
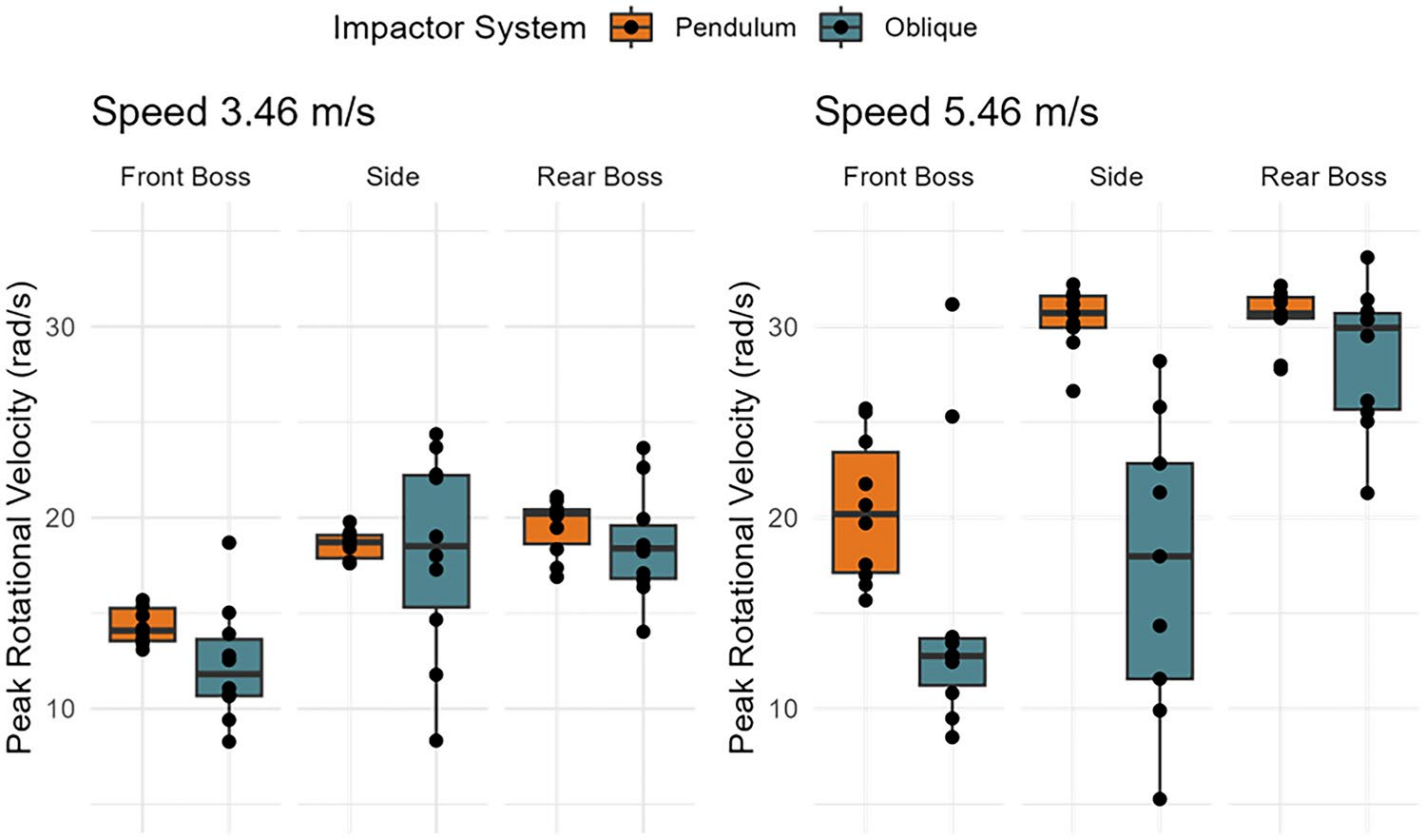
Distributions of peak rotational velocity between test system, impact location, and impact speed across all 10 helmets.

### Rotational Velocity

Test system (*p* < 0.0001), impact location (*p* < 0.0001), and impact speed (*p* < 0.0001) influenced rotational velocity measures (Fig. 6). On average, the oblique drop tower produced rotational velocities 4.34 rad/s [2.69, 5.98 rad/s] higher than the pendulum impactor. Adjusted R-squared values from the linear models of the pendulum compared to the oblique system suggested moderate associations between test systems for rotational velocity (Table 5).

**Table 5:** Adjusted R-squared values describing how much of the variance in one system is explained by the other system for Peak Rotational Velocity

Speed (m/s)	Model	Adj. R-squared	*p*-value
3.46	Oblique ~ Pendulum	0.474	< 0.0001
5.46	Oblique ~ Pendulum	0.154	0.0181

**Fig. 7:**
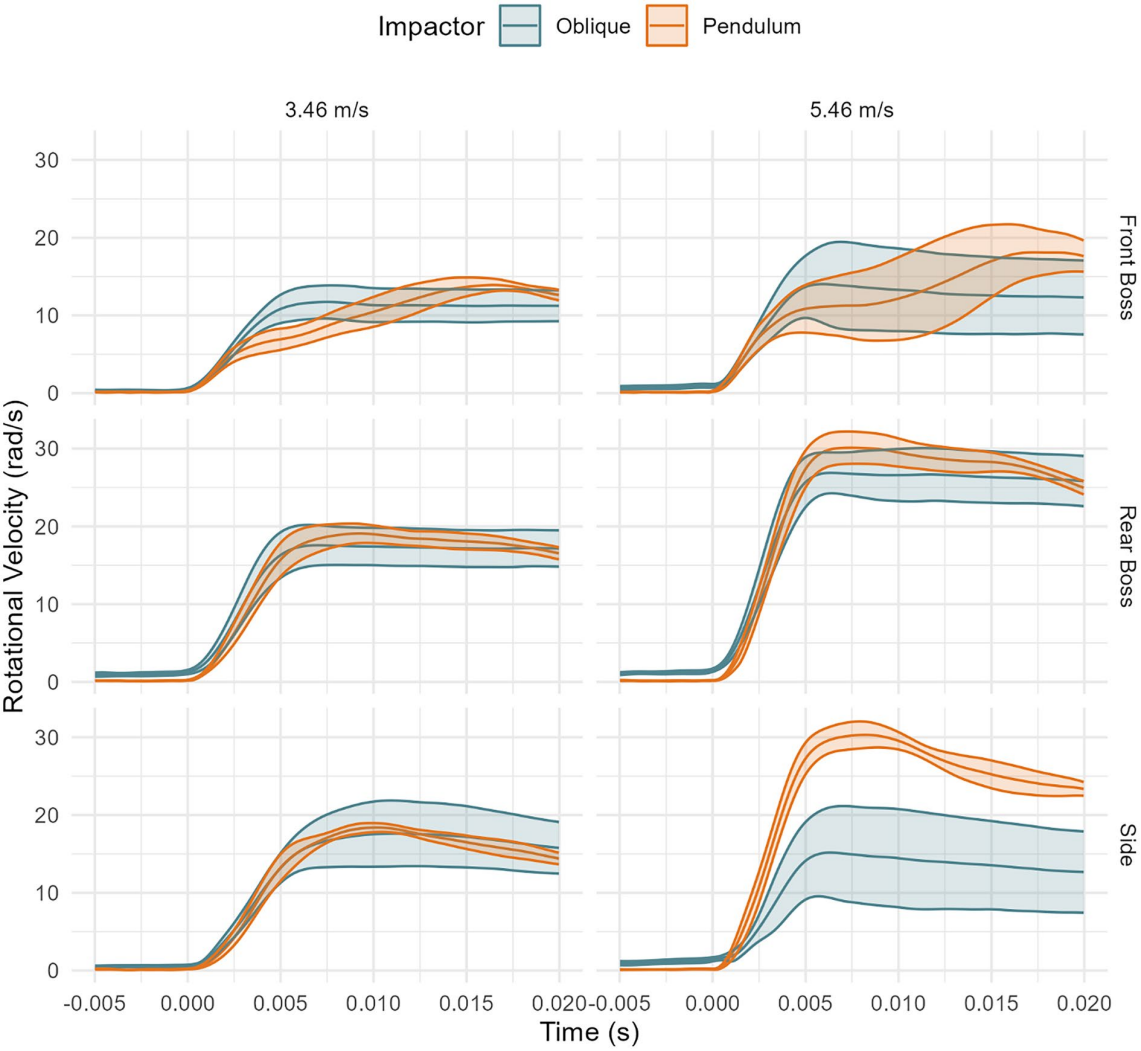
Rotational velocity time traces between systems and by location (mean and 95% confidence intervals). Oblique impacts had higher rotational velocity values.

The oblique system had a higher rotational velocity over the impact duration (Fig. 7). When comparing axis-specific traces, rotational velocities had similar traces, but the oblique system had a higher magnitude Appendix Figs. 15, 16, 17). The oblique drops had tri-axial rotation, while the pendulum had primarily X and Y rotations.

### Helmet Model Comparisons

The Charles Owen Sovereign and Casablanca NEU helmets produced the lowest average linear accelerations across all test systems and the La Martina Windsor and Armis Edge helmets produced the highest (Fig. 6). Comparing linear acceleration, moderate to strong correlations for helmet model rank order were observed between the test systems (Table 6). However, the Instinct Askari and KEP Cromo 2.0 helmets produced the lowest average rotational accelerations across both test systems, and the Krono Alpha and La Martina X-Volution helmets produced the highest (Fig. 6). Helmet model rank order strongly correlated the oblique and pendulum test systems for rotational acceleration (rho = 0.77, *p* = 0.0137).

**Table 6:** Spearman rank correlation coefficients (rho) for linear acceleration between test systems

Model	rho	p-value
Oblique ~ Pendulum	0.85	0.0035
Oblique ~ NOCSAE	0.75	0.0184
NOCSAE ~ Pendulum	0.62	0.0603

Comparing concussion risk, the Charles Owen Sovereign and Armis Vera helmets produced the lowest average concussion risks across test systems and the Krono Alpha and La Martina X-Volution helmets produced the highest (Fig. 6). Helmet model rank order showed a strong correlation between the oblique and pendulum test systems (rho = 0.82, *p* = 0.0068).

## Discussion

In this study, we evaluated how different helmet impact test systems influences headform kinematic response through the comparison of the 10 polo helmet models. These findings highlight differences and draw comparisons between helmet impact testing systems. We found that the impact response varied between test systems at matched impact speeds. Despite this variance, there was some agreement between test systems in the rank order of helmet models, particularly for linear than rotational acceleration. Furthermore, the linear and rotational acceleration ranges across different helmet models were substantial, translating to large differences in concussion risk between helmet models.

**Fig. 8:**
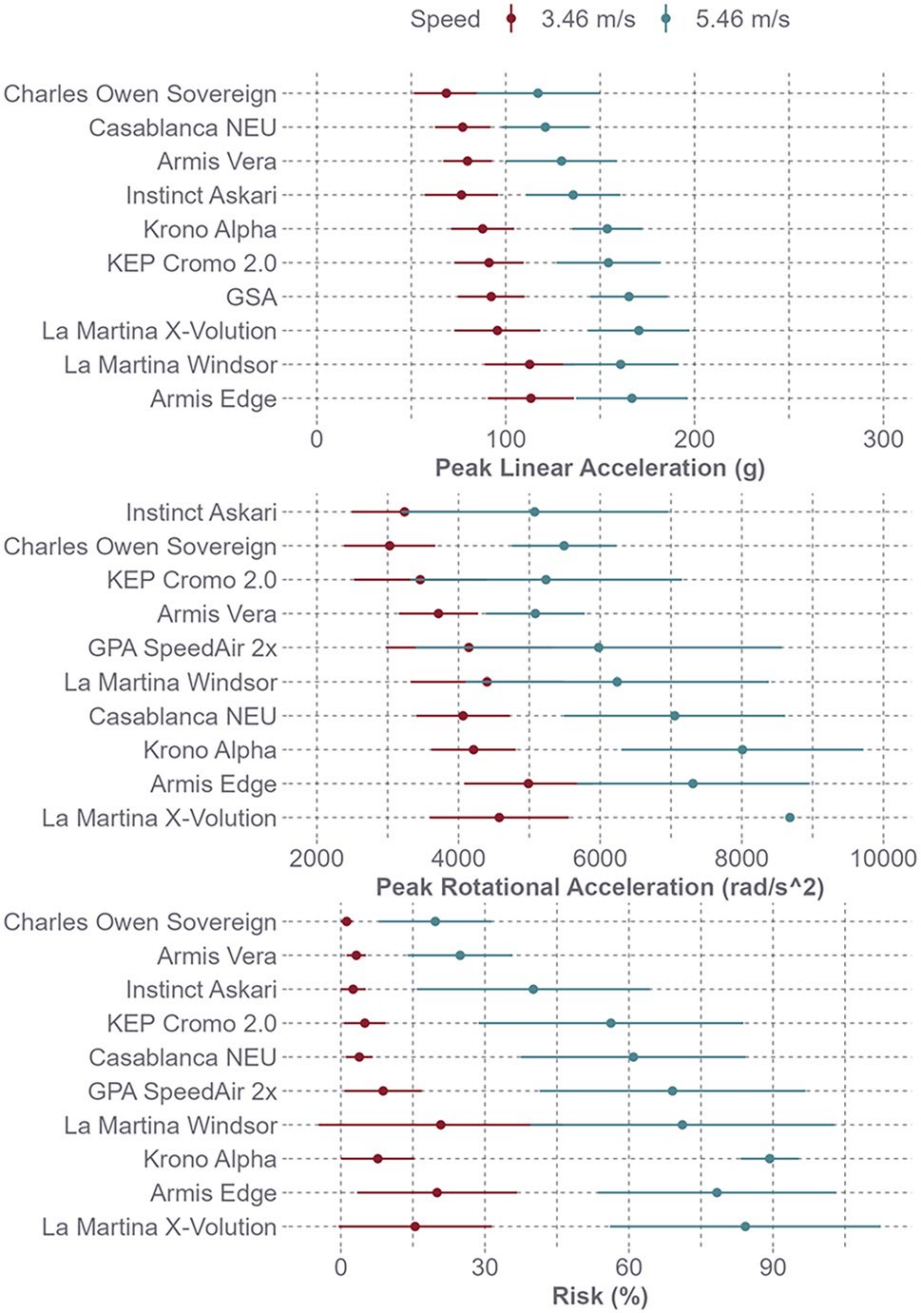
Mean and 95% confidence intervals for each helmet model across test systems by impact speed.

**Fig. 9:**
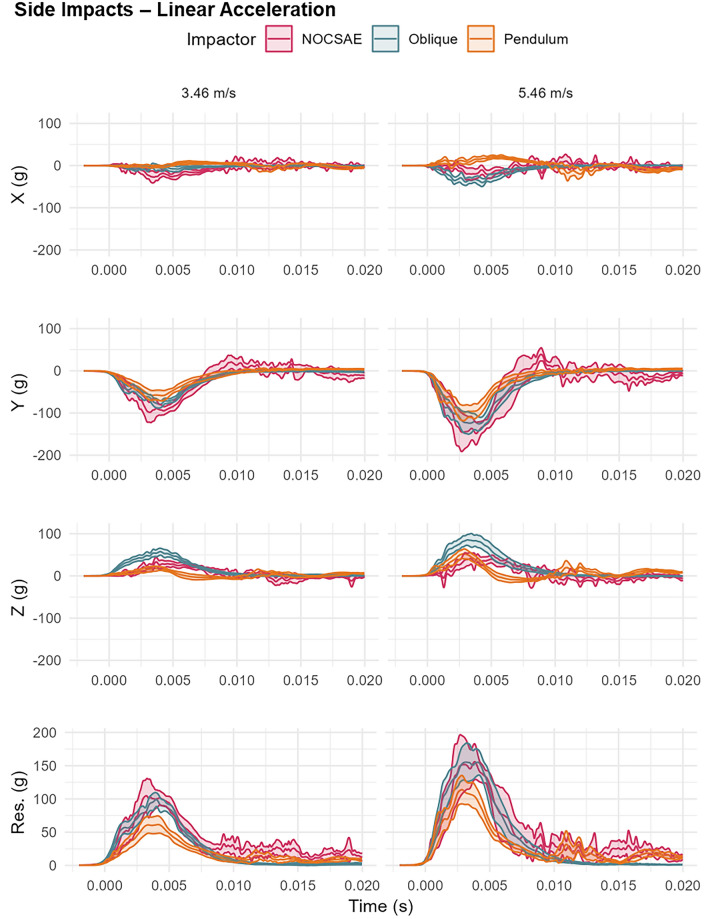
Linear acceleration time traces by axis-specific components for side impacts at low and high speed.

**Fig. 10:**
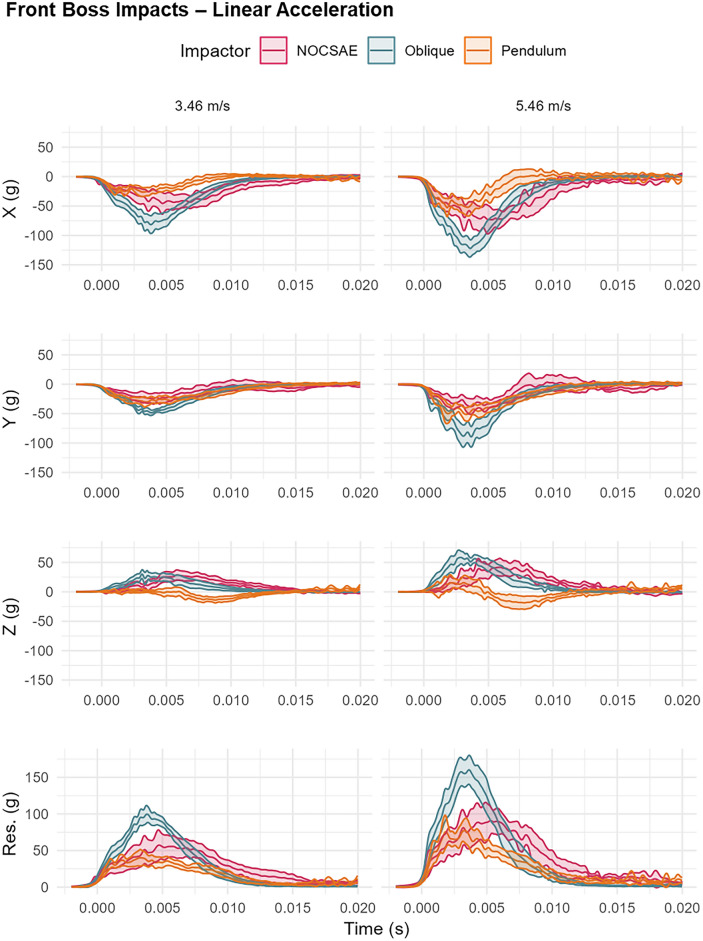
Linear acceleration time traces by axis-specific components for front boss impacts at low and high speed.

**Fig. 11:**
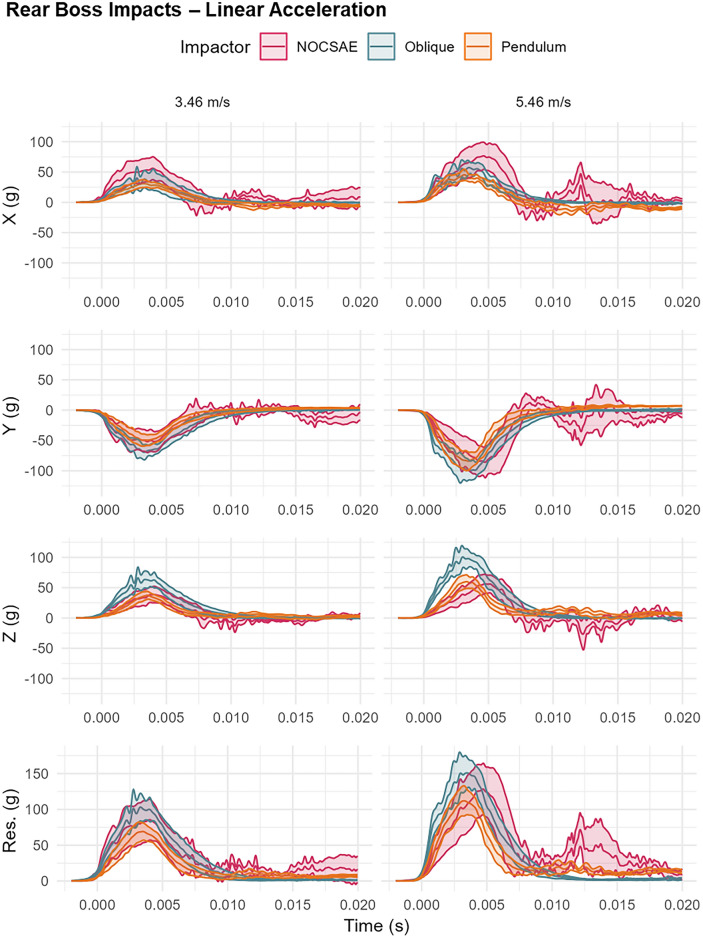
Linear acceleration time traces by axis-specific components for rear boss impacts at low and high speed.

Across test systems, linear acceleration responses showed similarities in the rise times and durations for the side and rear boss locations at both impact speeds (Fig. 3). Despite these similarities, there was a substantial range of linear acceleration values across different polo helmet models. For example, when comparing PLAs across helmet models for the 5.46 m/s tests (Fig. 8), we noticed that some helmet models generated PLAs at or below 100 g while other models nearly reached 200 g. This spread in PLA values was notable when also considering the nonlinear nature of the bivariate injury risk function used to estimate concussion risk. Both the pendulum and oblique systems identified the same top-performing helmet, whereas the NOCSAE drop system identified a different model. The difference in helmet ranking based on test systems depicts that the systems are not interchangeable when identifying a helmets ability to mitigate PLA (Table 6). The pendulum system recorded the lowest PLA, averaging 87.3 g [95% CI 75.9, 98.7 g]. In comparison, the NOCSAE system had an average PLA of 40.9 g higher than the pendulum system, while the oblique system had an average of 52.7 g higher. This finding is consistent with our first hypothesis that the pendulum would generate lower head accelerations. The NOCSAE and oblique systems, use a fixed impact surface, while the pendulum system allows the impactor to swing through the headform, maintaining contact throughout the impact. Consequently, the headform in the pendulum system does not strike an immovable mass, reducing the vertical translational forces that are more pronounced in the NOCSAE and oblique system. Furthermore, during a drop test, the head change in velocity will match the drop speed (or be greater if a rebound occurs). In contrast, during pendulum tests, the head's change in velocity will be less than the pendulum's impact speed, as the pendulum itself undergoes a change in velocity upon impact.

The oblique system showed an average PLA that was 11 g higher than that of the NOCSAE system. This difference arises because, in the oblique system, the headform strikes a metal anvil, causing the impact energy to be transferred back into the helmet or redirected toward the rotation of the headform. Conversely, the NOCSAE system includes a standard MEP pad and does not allow the headform to rotate due to its rigid attachment to the carriage. Although the oblique system represents a free-fall scenario, our results differ from previous findings, which found lower PLA for free-fall systems (flat and oblique) compared to a guided fixed system due to the difference in our impact surface [15, 17, 18]. Furthermore, while the NOCSAE and oblique systems had similar PLA ranges, the test systems were not correlated for helmet performance. Rank order of helmet performance measures varied across these systems, suggesting distinct behaviors for different helmet models. Unlike previous studies that suggested the NOCSAE-guided system represents a worst-case scenario compared to free-fall impacts, our findings indicate that these two systems exhibit distinct differences in impact kinematics, leading to variations in helmet response between systems.

Previous studies found that free-fall impacts increased concussion risk by 95% compared to guided falls, as they capture the rotational impact kinematics linked to head injury [15]. Due to the guided drop design, the NOCSAE system does not involve rotational impact kinematics, so PRA, PRV, and concussion risk were not assessed for this system. Both rotational test systems, the oblique and pendulum systems, identified the same helmets as top performers (rho = 0.77). However, the oblique system produced rotational accelerations that were, on average, 460 rad/s^2^ higher than those of the pendulum impactor. These findings are consistent with our second hypothesis that rotational kinematics would differ between the two impact test systems. The difference between test systems is due to the pendulum's linear driving force. Consequently, PRA in the pendulum system is more closely correlated with linear acceleration, as it maintains a controlled rotational velocity. This controlled velocity occurs because the headform accelerates to nearly the same speed as the pendulum while rotating about a pivot point below the CG. In the oblique system, however, linear and rotational accelerations are less correlated because the applied force is not aligned with the center of gravity but rather includes a tangential force vector on the helmet’s surface, and the system lacks a fixed rotational constraint. The eccentricity of these impacts can describe the difference in loading. Eccentricity quantifies the offset of loading to the CG of the headform [33]. Calculated for the side impact location, the pendulum system eccentricity was 2.76 cm [2.55, 2.98 cm] system compared to the oblique system eccentricity of 1.45 cm [1.21, 1.70]. The range in eccentricity around the mean for the side impacts occurs from each helmet's unique geometry and eccentricity will vary by impact location. This difference in eccentricity between systems indicates different loading of forces at impact, explaining the increase in PRA for the pendulum system compared to the oblique systems.

Although PRA and PLA are more closely correlated in the pendulum system than in the oblique system, a moderate association was observed between the two systems for rotational acceleration. However, when comparing time series data, we observed greater variation in the oblique impact traces. Additionally, the rotational acceleration time traces showed distinct axis-specific component traces for the oblique and pendulum systems (Appendix), primarily due to the neck constraint in the pendulum setup. Notably, clear kinematic differences between the two systems were observed, particularly in scenarios where impacts at identical headform locations produced rotations in opposite directions. For instance, at the front boss location, the directions of rotation about both the x- and y-axes differed between the pendulum and oblique systems. These directional differences are attributed to the neck constraint and sustained contact between the helmet and impactor face throughout the pendulum impacts. Specifically, at the front boss, the relatively small rotational velocities about the x- and y-axes, along with larger 95% confidence intervals observed in the oblique system, indicate that the headform experienced limited induced rotation in these axes due to it largely rebounding off the anvil. In contrast, the pendulum impacts exhibited larger and more consistent rotations about the x- and y-axes because the headform is constrained and the impactor face maintained continuous contact with the helmet throughout impact. Similarly, at the rear boss location, rotation in the oblique system predominantly occurred about the negative x-axis and positive z-axis, whereas in the pendulum system, rotation occurred primarily about the negative x-axis and negative y-axis. This divergence in rotational behavior again reflects the absence of neck constraints and the tendency of the headform to rebound from the anvil in the oblique system. Conversely, the pendulum system’s neck constraint and constant helmet-to-impactor face contact throughout the impact dictated the rotational direction toward the negative x- and y-axes. These differences underscore the critical influence of system-specific constraints and highlight that impact responses should be compared to real-world events to ensure that systems are representative.

The difference between test systems also correlates to differences in concussion risk. Concussion risk was calculated only for the oblique and pendulum systems, as these systems account for PRA, whereas the NOCSAE system lacks PRA and would underpredict concussion risk. Helmet model rankings showed a strong correlation between the oblique and pendulum test systems (rho = 0.82, *p* = 0.0068). Both rotational test systems identified the same helmets as top performers in minimizing concussion risk. Specifically, the Charles Owen Sovereign and Armis Vera helmets produced the lowest average concussion risks across test systems. Conversely, the two systems did not agree on the worst-performing helmet. The pendulum system identified Krono Alpha compared to the oblique system, identifying Armis Edge as the worst-performing helmet. The moderate correlation and difference in helmet rank produced by the two systems indicates that each system has unique kinematics that relate to different real-world impacts.

Both the head impacts produced by pendulum and oblique systems are possible in polo, and each provides unique information on the impact response of a helmet. Therefore, there may be value in a comprehensive test protocol that includes multiple impact test systems. This study is limited in that it also did not collect or evaluate any on-field data of polo players, and future work should quantify the boundary conditions of head impact injuries in polo to determine test methods representative of polo head impacts for helmet testing results to be representative of player risk.

Out of the tested models, four were NOCSAE-certified. NOCSAE-certified helmets demonstrated superior performance on all test systems, producing lower linear and rotational accelerations than non-NOCSAE-certified helmets. On average, these helmets generated linear accelerations 29.7 g [19.1, 40.3 g] lower than their non-certified counterparts. Additionally, NOCSAE-certified helmets produced rotational accelerations that were 992 rad/s^s^ [125, 2109 rad/s^s^] lower on average than non-certified helmets. In terms of concussion risk, NOCSAE-certified helmets exhibited a 55% relative risk reduction compared to non-NOCSAE-compliant helmets.

Other study limitations include head kinematics were compared across different test systems using only polo helmets. Although general trends may hold true for other helmet types, the results presented in this work are specific to helmets used in polo and not extrapolated to helmets from other sports and industries. Another limitation is that impact speeds were restricted to those outlined in the NOCSAE standard. It is possible that other impact speeds could have been selected to represent polo players who were ejected cleanly from their horses or used evasive maneuvers to mitigate fall speed. Another study limitation is that the impact surfaces were selected to be best matches between impact systems, which does not account for the variance in head impact surfaces observed in polo accidents. Therefore, when reconstructing polo head impacts, future studies should consider the effects of surface friction and stiffness. Fourth, multiple impact locations were selected to maximize the number of tests that could be conducted on individual helmet samples despite polo helmets being considered single-use style helmets. However, it should be noted that the centers of each impact location were spaced apart sufficiently to ensure that the damage zones did not overlap. Another limitation is the front boss location on the pendulum system could induce axial loading on the Hybrid III neck, which could have confounding effects on the results for the front boss impact location. When axially loaded, the Hybrid III neck results in acceleration responses that can deviate from real-world impact responses [34, 35]. Therefore, this must be considered when determining the test device used to reconstruct the impacts. The last limitation of this study is that the concussion risk function used does not account for the brain’s directional tolerance to head acceleration.

This study quantified the impact response of different helmet impact test systems by comparing current polo helmets across the varying loading conditions. This study demonstrates that at matched impact speeds, responses differed between test systems. However, there was some correlation and agreement in the rank order of helmet performance across systems. Furthermore, variations in acceleration between helmets led to substantial differences in concussion risk across helmet models. While the rotational test systems differed, both types of impacts could occur in polo, suggesting that each system may be valuable in assessing the protective capabilities of helmets. Overall, these findings provide a framework for comparing headform responses across various loading conditions. When selecting a test system to evaluate helmets for a specific sport, it is essential to consider the relevant impact conditions and loading patterns to ensure that laboratory tests accurately reflect real-world scenarios. Additionally, a combination of test systems may be necessary to best represent real-world impact conditions and determine overall helmet performance.

## Figures and Tables

**Fig. 12:**
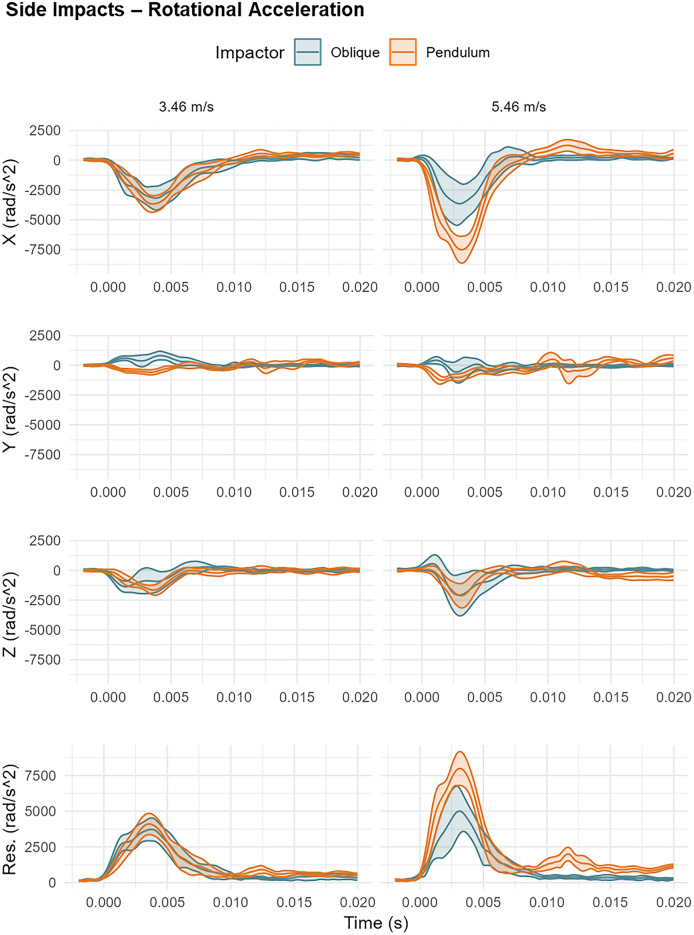
Rotational acceleration time traces by axis-specific components for side impacts at low and high speed.

**Fig. 13:**
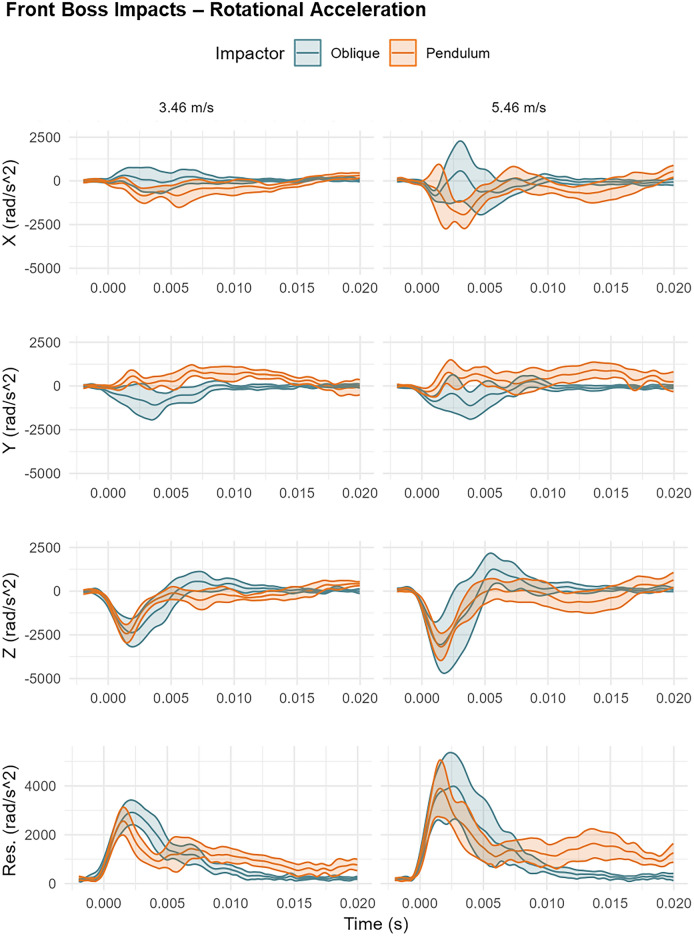
Rotational acceleration time traces by axis-specific components for front boss impacts at low and high speed

**Fig. 14:**
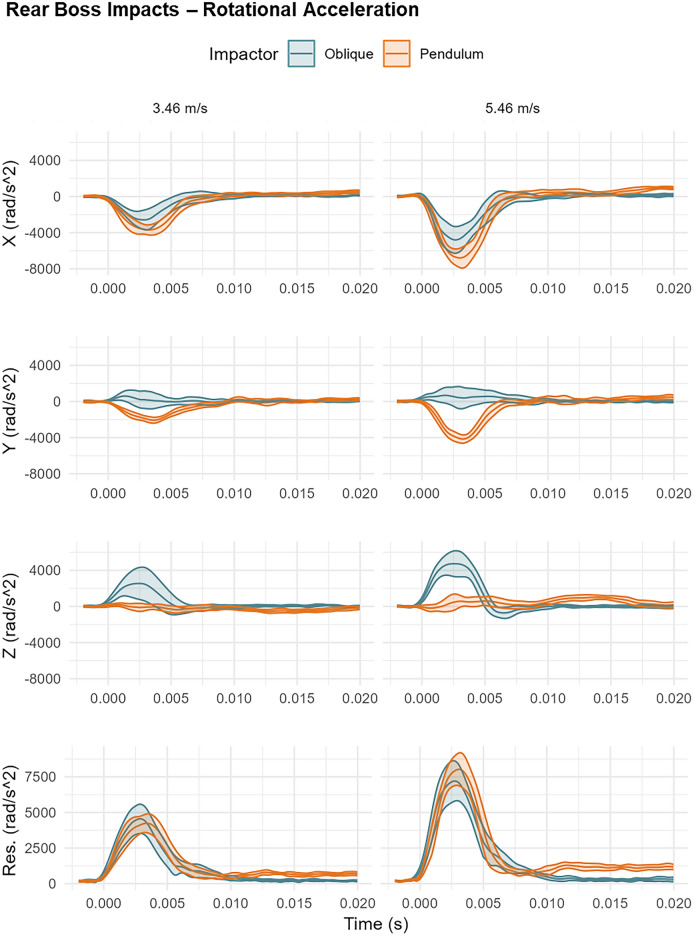
Rotational acceleration time traces by axis-specific components for rear boss impacts at low and high speed

**Fig. 15:**
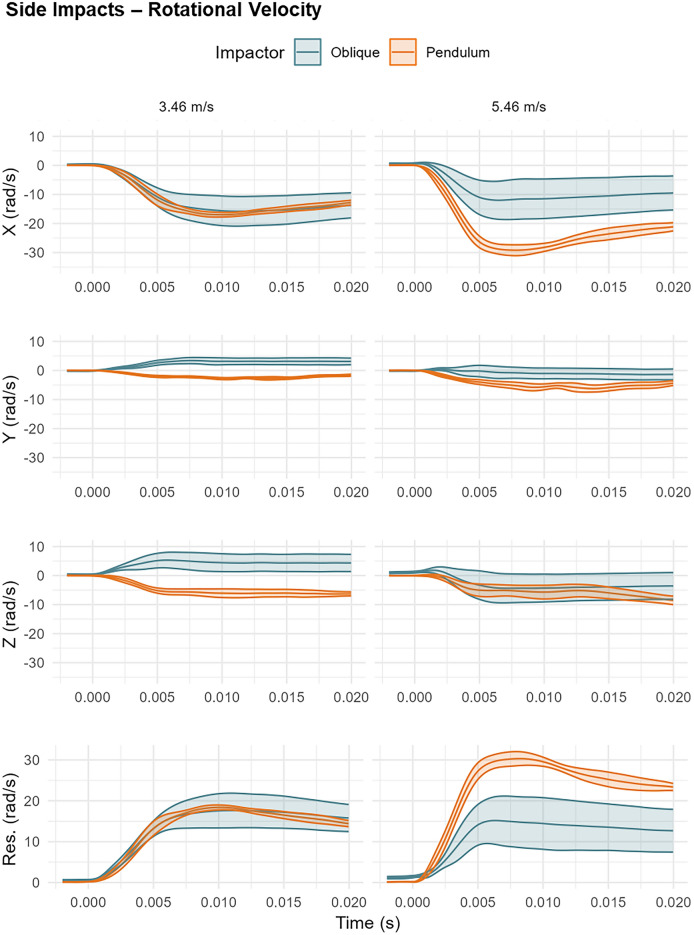
Rotational velocity time traces by axis-specific components for side impacts at low and high speed.

**Fig. 16:**
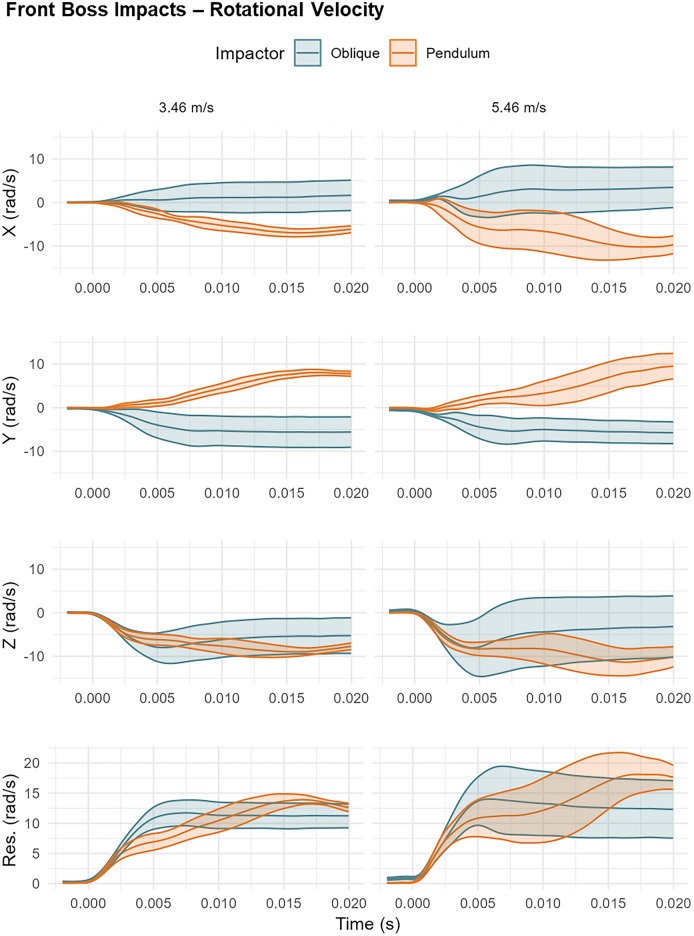
Rotational velocity time traces by axis-specific components for front boss impacts at low and high speed

**Fig. 17:**
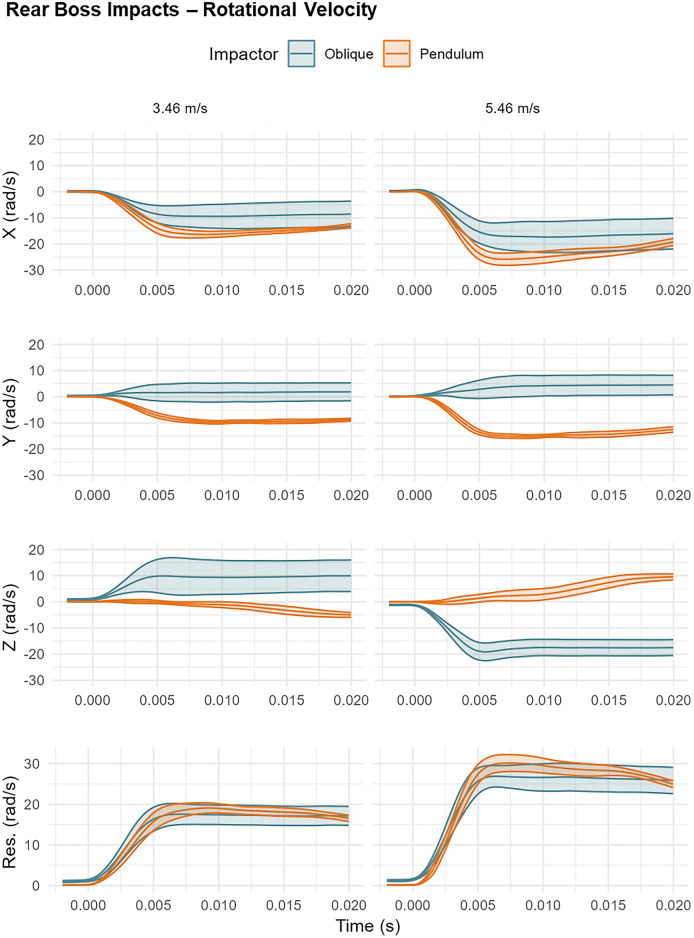
Rotational acceleration time traces by axis-specific components for rear boss impacts at low and high speed

## Appendix A

### Impact Locations

See Appendix

**Table 7:** Impact location information for the NOCSAE system (right-sided impacts)

Location	Rotator position	Stem position	Nose position
Front boss	1	1	Southeast
Side	1	2	East
Rear boss	6	3	Northeast

Note that the position descriptions are based on the NOCSAE position system outlined in Appendix 2 of ND-001. The numbers denote assigned through-holes in the rotator and stem components for inserting a pin, while the compass directions indicate the direction in which the NOCSAE nose is pointing relative to an overhead view of the helmeted headform when mounted on the assembly

Tables 7,

**Table 8:** Impact location information for the pendulum system (right-sided impacts)

Location	Y Translation (cm)	Z Translation (cm)	Y Rotation (°)	Z Rotation (°)
Front boss	0	− 2.7	− 45	− 55
Side	− 2	5.3	− 20	− 100
Rear boss	− 2.5	6.8	0	− 125

Note that all measurements are made using the SAE J211 coordinate system in relation to a zero position defined by (1) the headform facing the impactor (0° y and z-rotations) and (2) the impactor center aligned with the intersection of midsagittal and basic planes on the headform face (bridge of nose). For each location, the x-position was set such that the helmet surface barely contacts the impactor face when the pendulum arm is in a neutral position

8, and

**Table 9:** Impact location information for the oblique system (right-sided impacts)

Location	X Rotation (°)	Y Rotation (°)	Z Rotation (°)
Front boss	13.1	− 4.2	80
Side	− 30	− 20	− 170
Rear boss	− 25	40	170

Note that X and Y are defined according to the SAE J211 coordinate system whereas Z is defined by projecting the midsagittal plane of the NOCSAE headform onto the halo. The halo is inscribed in 5° increments, with 0° corresponding to the headform facing the drop tower and positive angles corresponding to a clockwise rotation

9.

### Axis Trace Figures

See Appendix Figs. 9, 10, 11, 12, 13, 14, 15, 16, and 17.
